# Book Notices

**Published:** 1860-07

**Authors:** 


### Book Notices.

*The reader is specially invited to read all the following notices, for many of them are practical, and all are useful.*

Balliere Bros., 440 Broadway, New-York, have issued a comely 12mo of 318 pp., $1, entitled, *A Knowledge of Living Things, with the Laws of their Existence*, by Dr. A. N. Bell, late Surgeon in U. S. Navy, and now physician to Brooklyn City Hospital. It is a volume which may be read with absorbing interest by every student of nature, and by every scholar, while it abounds with information practically useful for all classes of readers.

*The Human Voice.* Fowler & Wells. 30 cents. By Rev. W. W. Cazalet, A.M. Cantab. Its right management in speaking, reading, and debating, including the principles of true eloquence; contains a large amount of useful information to all public speakers and singers.

*The Chemist and Druggist.* A monthly trade circular, 24 Bow Lane, London. $2 a year, single numbers, 12 cents. Contains a vast amount of information for apothecaries, druggists, medical and surgical instrument dealers, etc. etc. New-York office, C. F. A. Henricho, 150 Broadway.

*Patients' and Physicians' Aid.* By Dr. E. M. Hunt; 365 pp. 8vo. with copious index. Published by C. M. Saxton, Barker & Co., New-York, and H. H. Bancroft & Co., San Francisco. Showing "how to preserve health, and what to do until the doctor comes," a kind of information greatly needed in multitudes of families in the country. There is not a household in the land which should be without a plain, reliable book of this kind, it would prevent much mental and bodily suffering, and often save life.

*Thermometers.*—V. Beaumont, 175 Centre street, New-York, has patented a circular, dial-plate, metallic Thermometer, where no mercury is visible; a long index, like that of the minute-hand of a watch, shows the degree of heat or cold by Fahrenheit's scale parallel with the centigrade. Its delicacy is such, that laying it on the hand for an instant, changes the degree. It can be easily carried in the vest-pocket, and is altogether the best pattern of a thermometer we have ever seen.

*The Water-Cure Journal.* Monthly, $1 a year, New-York. Fowler & Wells have done much for the promotion of temperance, cleanliness, and hygiene. But why don't it keep its temper? A madman always loses the battle, whether in the "Ring," on the Forum, or on the Tripod. Look how it blazes away at some person whom it supposes advocates tight-lacing. Now tight-lacing is a prime remedy for maniacs; nothing so calms them down as a strait-jacket!

"One of our allopathic medical journals has advocated tight-lacing as a preventive and a curative measure for consumption; a proposition so self-evidently absurd, so ridiculously silly, would hardly call for serious refutation, were it not put forward and urged and reïterated with a show of anatomical and pathological knowledge just sufficiently plausible to deceive the ignorant and mislead the un thinking. The author of this outrageously foolish notion is not only the editor of a medical journal, but the author of a work on consumption; and however nonsensical his teachings may seem to us, and are of themselves, we are bound to regard him as earnest and honest in his opinions. It can not be possible, at least we will not believe it, that he misleads the people intentionally for the sake of the professional perquisites and advantages. We treat his lucubrations on this subject with more attention, also, because they have been extensively copied and circulated through the newspapers of the country without note, comment, or dissent, which is equivalent to their indorsement." Verily!

We thought that hydropathists contended that if a man would live on bran, berries, and fruits, would soak in cold water every day, and open a canal for it through the body to slosh out all its impurities, he would become as active and unkillable as a cat; his heart all the time chock full of love towards every body and the rest of mankind too, while the mind would be habitually as clear as a bell and as calm as a summer evening. Rather think there is a screw loose somewhere. Either the theory won't work, or the editor won't practice it, and both are going to seed. Hope not, for few papers contain more good things when its editor is oblivious of the fact that there is a drug or an allopathic doctor in existence.

*New-York Teacher.* Albany; $1 a year, 48 pp. There are several articles in this valuable monthly for May, which merit a wide distribution. Physical Culture, (from the *Massachusetts Teacher*, Boston,) Classical Training, by Henry C. Mitchell, Necessity of Teaching Children to Think, Aids to Science, Spelling and Writing, At what Age and how many hours for Schooling Children, by Luther Haven, of Chicago, The Family.

The *Western Watchman*, of St. Louis, is one of the handsomest sheets among our religious exchanges, and we always open it with interest. Neither it nor the *Watchman and Reflector*, of Boston, nor the *True Union*, of Baltimore, nor the *Examiner* or *Chronicle*, of New-York, ever pass from our hands without the wish to read them more thoroughly. These are all Baptist publications, and are well worthy of a large patronage among that very numerous denomination of Christians.

No lover of the beautiful in art and mind culture, can open the fair pages of the *Musical World* ($2 a year, New-York) without finding something to feed upon; something elevating and refining. Its last page of reading-matter, made up of short sentences, must be the work of a cultivated mind and heart.

*The Louisana Baptist*, at Mount Lebanon, we always receive with interest; we trust it receives the patronage which it merits among that large body of Southern Christians.

*The Banner of the Cross*, of Philadelphia, seldom passes from our hands, without the wish for leisure to read more of it. It is really a family paper which can not fail to benefit those who read it regularly.

We would like to know that the *Witness*, of Indianapolis, is as liberally patronized as a paper of its merit and influential position deserves. We have pleasant memories of beautiful Indianapolis.

*Bare Necks, Arms, and Legs of Children*, their evil effects; see June Number, 1859.

*A Wonder.* A lady correspondent writes: "My mother has taken from the left side, near the heart, a piece of a needle an inch in length, which broke off near the center of the palm of the left hand fifteen years before. For nearly all that time it has caused untold sufferings, feeling the pain at intervals in arm, back, shoulders, spasmodic anguish at the heart and many other symptoms indescribable." Similar cases are given in standard medical works. It is a rule in the animal economy that when any foreign substance is introduced into the living body, nature gives no rest until it is expelled ; hence the utmost care should always be given to extract every wounding material, whether of wood, glass, or metal ; the sooner it is done after an accident the better. This piece of needle traveled up the arm, over the arm-pit, along the ribs and thence outwards.

*Lung and Life-Preservers.* As to our May article on Wind and Lungs, we merely add: If a person wishes to be benefited, let him purchase any where an India-rubber life-preserver to be inflated with air. Let a healthy man of your own age, weight, and hight see how many breaths it requires for him to fill it to the utmost. If he does it at one full expiration, and you can not, your lungs are working imperfectly. If it requires two full breaths for him, and three for you, then one third of your lungs are inoperative, and you should practice its inflation several times a day, or run a certain distance and back thrice a day with the mouth closed until the life-preserver can be easily filled by you in the same number of breaths as by your friend. Forty cubic inches to a pint is the standard adopted by English physicians for lung-measurement. That need not be taken into account. The question is, can you fill a life-preserver with as few full breaths as a man of the above conditions ; if not, you are in danger of dying of consumption sooner or later, and will certainly do it (unless this ill condition is rectified) if you do not perish by violence.

Right pungently does the veteran editor of the *American Medical Gazette* apostrophize his readers on the occasion of so slim an attendance on the sanitary lecture of E. Y. Robbins, Esq., of Boston :

" New-Yorkers are too busy in paying their devotions to the almighty dollar, to attend to such insignificant matters as life and death, health and sickness, etc. ; they can not be persuaded from the opera, the theater, the ball-room, the negro-minstrels, and the grog-shops, to learn how to prevent pestilence or lessen mortality. Alas ! that Wendell Phillips & Co., lecturing on the everlasting Negro, and teaching treason, should draw crowds of listening dupes, while lessons of philosophy and wisdom from eloquent lips should be repeated to empty benches. He who would attract the multitude should play the fool, make gorgeous shows of magic-lanterns, administer laughing-gas, or shoot off fire-crackers, for nonsense and humbug are in the ascendant."

That's true, Doctor, but people must have fun, and they will crowd where there is the most of it. Phillips is full of mirth and wit. People go to hear him because they know they will get a good number of tremendous laughs, while they might not, and many of them do not, value his principles or his logic beyond the worth of a pewter button. They will command the largest audiences in a New-York community who will abound most in mirth and wit, the subject being secondary, Let public speakers, reformers, and humanitarians remember this ; for after the severer occupations of business and labor during the day, the body demands repose and the mind yearns for diversion, which is its rest, and will have it, and when pleasurable, so much the more satisfactory, efficient, and recreative.

*Life Illustrated* calls us to task for heading an article "Protest against Early Rising." It was done for us by some lazy editor. It well says that the title alone would give a first impression which would be injurious as well as untrue. We both agree that under all circumstances a plenty of sleep is essential to health, and that all should practice early rising, and begin the day at least at sunrise with mind, body, and brain fully rested and renovated.

*Health and Disease.* A correspondent from the " Old North State," says, of this publication: "I am so pleased with its just and sensible views, that I feel I am doing a real good to every one whom I can induce to get a copy." A lawyer of this city said of it to a friend: " I have derived more instruction, and have been made better acquainted with the nature of health and disease by reading Dr. Hall's book on that subject, than from all the books read in a lifetime." The third edition of this book having been called for within nine months after its first issue, is evidence of its appreciation. It is sent, post-paid, for one dollar.

A Westoner writes of the JOURNAL OF HEALTH: " I am well pleased with it. You talk more to the point than any man I have ever read; in other words, you have more white-oak sense than any one I have found. I mean by that, more good mother-wit." He wanted us to *give* him a book. We did!

*Milk.* Dr. Gardner, of this city, a connoisseur in milk, says that he had used the milk of the Rockland Co. Association, and knew it to be good; had recommended to various families, and of all the children brought up on it from birth, under his eye, not one had failed to do well and gain flesh. Dr. Clark, of Newark, and the veteran Dr. Reese, with the Hon. Erastus Brooks, all of whom have used the milk, corroborated the statements of Dr. Gardner. We ourselves know that it is pure milk from farm-house fed cows. 146 E. Tenth st., New-York.

The editor of the *Hightstown Village Record* pokes fun at us in this style:

" RULES FOR SLEEP—AN IMPROVEMENT ON DR. HALL.—1st. As soon as you are in bed, have Bridget hand the wash-bowl to you. Then place it immediately beneath the small of your back, and you will immediately sink into a calm slumber. It should not remain in that position long enough to produce stupor.

" 2d. Try to think of something you can't remember; the more you can't think of it, the sleepier you will get.

" 3d. Let John or Phineas pour ice-water down the sleeve of your shirt for an hour or two, while he holds a lump of assafœtida to your nose.

" 4th. Count two millions slowly and deliberately. You will certainly be asleep before you have counted that number.

" 5th. Hold a wire against the nerve of your tenderest tooth. This is infallible —patent applied for.

" 6th. Have your back gently smothed with a curry-comb.

" 7th. Try and fix your mind on one thing; if that don't succeed, fix it on two."

In examining the exchanges brought to us on Saturday last, there were seventeen articles from our pen, which shows very fairly the partiality of our editorial brethren. A few days earlier we took up a paper which contained five columns of our handiwork, but looking for the " credit," we found "nary red." A sixth column, however, in another part of the paper, seemed to indicate that the editor had some recollection of the fact that there was such a publication as HALL'S JOURNAL OF HEALTH. But who cares for credit as long as the more important thing is done, that of disseminating what is useful and true? Let us all join in the song:

" Row, brothers, row,
The tide runs fast;
The rapids are near,
And the daylight's most past,"

and " Work while the day lasts."

*Hall-y.* George J. Hall, of Hall County, Georgia, writes to Dr. Hall to send him Hall's Journal of Health for 1860.

A gentleman who has been married for perhaps two weeks, at a guess, writes: "We, (that is, self and wife,) the only two members of as happy a family as ever was, can not do without the Journal of Health." He wants further to know what is the cause and cure of palpitation of the heart. The first kiss from "sweet seventeen" will give it to any body; the "mitten" will cause its very speedy subsidence. It is an unimportant symptom of dyspepsia.

A subscriber to the Journal of Health, and to the Fireside Monthly, writes: "'Dr. Hall says so,' is thought the *finale* in any argument or recommended in my family."

That excellent and standard weekly, *The Country Gentleman*, quotes from Lavater, "Young women who neglect their toilette indicate in this very particular a disregard for order, a deficiency in taste, and the qualities which inspire love. The girl of eighteen who does not desire to please in so obvious a matter as dress, will be a slut and probably a shrew at twenty-five;" and that there is more pleasure in giving than in receiving, especially as to medicine, kicks and advice; and further: "Most persons are particularly spiteful against the follies in others which they themselves have."

Missing numbers of the Journal are supplied to subscribers for six cents. All subscriptions begin with January last.

